# Development of an Inactivated H7N9 Subtype Avian Influenza Serological DIVA Vaccine Using the Chimeric HA Epitope Approach

**DOI:** 10.1128/Spectrum.00687-21

**Published:** 2021-09-29

**Authors:** Zhihao Sun, Qiuxia Wang, Gang Li, Jingzhi Li, Sujuan Chen, Tao Qin, Hongwei Ma, Daxin Peng, Xiufan Liu

**Affiliations:** a College of Veterinary Medicine, Yangzhou University, Yangzhou, Jiangsu, China; b Suzhou Institute of Nano-tech and Nano-bionics, Chinese Academy of Sciences, Suzhou, Jiangsu, China; c Jiangsu Co-Innovation Center for the Prevention and Control of Important Animal Infectious Disease and Zoonoses, Yangzhou, Jiangsu, China; d Jiangsu Research Centre of Engineering and Technology for Prevention and Control of Poultry Disease, Yangzhou, Jiangsu, China; e Joint International Research Laboratory of Agriculture and Agri-Product Safety, The Ministry of Education of China, Yangzhou, Jiangsu, China; Texas A&M University

**Keywords:** avian influenza virus, H7N9 subtype, DIVA vaccine, epitope, peptide microarray

## Abstract

H7N9 avian influenza virus (AIV) is an emerging zoonotic pathogen, and it is necessary to develop a differentiating infected from vaccinated animals (DIVA) vaccine for the purpose of eradication. H7N9 subtype AIV hemagglutinin subunit 2 glycoprotein (HA2) peptide chips and antisera of different AIV subtypes were used to screen H7N9 AIV-specific epitopes. A selected specific epitope in the HA2 protein of H7N9 AIV strain A/Chicken/Huadong/JD/17 (JD/17) was replaced with an epitope from an H3N2 subtype AIV strain by reverse genetics. The protection and serological DIVA characteristics of the recombinant H7N9 AIV strain were evaluated. The results showed that a specific epitope on the HA2 protein of H7N9 AIV, named the H7-12 peptide, was successfully screened. The recombinant H7N9 AIV with a modified epitope in the HA2 protein was rescued and named A/Chicken/Huadong/JD-cHA/17 (JD-cHA/17). The HA titer of JD-cHA/17 was 10 log_2_, and the 50% egg infective dose (EID_50_) titer was 9.67 log_10_ EID_50_/ml. Inactivated JD-cHA/17 induced a hemagglutination inhibition (HI) antibody titer similar that of the parent strain and provided 100% protection against high-pathogenicity or low-pathogenicity H7N9 AIV challenge. A peptide chip coated with H7-12 peptide was successfully applied to detect the seroconversion of chickens infected or vaccinated with JD/17, while there was no reactivity with antisera of chickens vaccinated with JD-cHA/17. Therefore, the marked vaccine candidate JD-cHA/17 can be used as a DIVA vaccine against H7N9 avian influenza when combined with an H7-12 peptide chip, making it a useful tool for stamping out the H7N9 AIV.

**IMPORTANCE** DIVA vaccine is a useful tool for eradicating avian influenza, especially for highly pathogenic avian influenza. Several different DIVA strategies have been proposed for avian influenza inactivated whole-virus vaccine, involving the neuraminidase (NA), nonstructural protein 1 (NS1), matrix protein 2 ectodomain (M2e), or HA2 gene. However, virus reassortment, residual protein in a vaccine component, or reduced vaccine protection may limit the application of these DIVA strategies. Here, we constructed a novel chimeric H7N9 AIV, JD-cHA/17, that expressed the entire HA protein with substitution of an H3 AIV epitope in HA2. The chimeric H7N9 recombinant vaccine provides full clinical protection against high-pathogenicity or low-pathogenicity H7N9 AIV challenge. Combined with a short-peptide-based microarray chip containing the H7N9 AIV epitope in HA2, our finding is expected to be useful as a marker vaccine designed for avian influenza.

## INTRODUCTION

The emergence of the novel H7N9 avian influenza virus (AIV) in March of 2013 has triggered five epidemics of human infections in China (1–4). There were 1,568 laboratory-confirmed human cases of H7N9 virus infection, including 616 deaths (a case fatality rate of 39%) (5). It raises concerns about the pandemic threat of the H7N9 virus to humans and whether this virus can potentially become more invasive to humans. Before the 2017 epidemic, the H7N9 virus circulating among chickens in China was classified as a low-pathogenicity AIV (LPAIV), and infection was asymptomatic in chickens (2). Importantly, the recently emerged H7N9 AIV that caused outbreaks in chickens was a highly-pathogenic AIV (HPAIV), leading to a large number of chicken deaths in several provinces of China (6), and the H7N9 HPAIV infection was identified in two patients in Guangdong province (7). Although there is no indication of human-to-human transmission, the potential for a pandemic is of great concern.

A recombinant, bivalent, inactivated avian influenza vaccine comprising the Re-8 strain of the H5N1 subtype and the Re-1 strain of H7N9, generated by reverse genetics based on the internal gene backbone of the egg-adapted, high-yield A/Puerto Rico/8/1934 (H1N1) virus, has been initially authorized by the Ministry of China, providing strong technical support to carry out effective prevention and control of H7N9 avian influenza. The isolation rate of the H7N9 virus in poultry dropped by 93.3% after vaccination (8). However, the continued presence of vaccine pressure and the rapid development of the live poultry trade may provide suitable conditions for variation of AIV, resulting in the emergence of sporadic infection in immunized chicken (9–11).

For pathogen eradication, vaccine design should preferably comply with the principle of serological differentiation of infected from vaccinated animals (DIVA) to distinguish infected animals from vaccinated ones (12). Several different DIVA strategies have been proposed for avian influenza. The heterologous neuraminidase (NA) vaccine strategy employs an inactivated AIV containing a similar subtype of hemagglutinin (HA) but a different subtype of NA compared to those of the outbreak strain (13), which allows screening for infection by determining heterologous anti-NA antibodies using an indirect immunofluorescence assay (13) or a conventional neuraminidase inhibition (NI) test (14). However, viruses that emerge by reassortment can potentially defeat the purpose of a DIVA strategy if the new virus contains an NA gene similar to that in the vaccine. Nonstructural protein 1 (NS1), a nonstructural protein that is only detectable in infected cells and not in packaged virions (15), can also be a DIVA antigen for the conventional inactivated whole-virus vaccines (16–18). However, commercial unpurified inactivated whole-virus vaccines induce low levels of NS1 antibodies (19). The relatively invariable nature of the matrix protein 2 ectodomain (M2e) protein across AIV strains and the abundance of the M2e protein on the surface of infected cells despite being low in copy number in a mature virion suggest that M2e protein could be a sensitive, specific, and universal DIVA antigen for conventional inactivated whole-virus vaccines (20, 21). However, it is difficult to determine subtype-specific antibodies among influenza A virus infections.

Hemagglutinin subunit 2 glycoprotein (HA2) is the more conserved region between the two HA cleavage products (HA1 and HA2) (22, 23) and has been suggested as another potential target for the DIVA tool even if it is a weak natural immunogen (24, 25). Moreover, some studies have shown that homologous-strain vaccination still provides by far the most optimum protection against virus infection, as antigenic relatedness is a significant factor in determining the level of protection induced by vaccination (26, 27). In this study, a peptide with 20 amino acids (aa) from HA2 of H7N9 AIV was identified as a specific epitope for H7N9 AIV, and then a DIVA H7N9 AIV vaccine candidate expressing chimeric HA by replacement of the H7N9 AIV epitope with one from H3 subtype AIV was developed. This vaccine protected chickens from H7N9 LPAIV and HPAIV challenge. Importantly, the DIVA aspect of the vaccine was confirmed by confirming the absence of antibodies against the 20-aa peptide from H7N9 AIV.

## RESULT

### Identification of an epitope containing a short peptide specific for the H7N9 virus.

**FIG 1:**
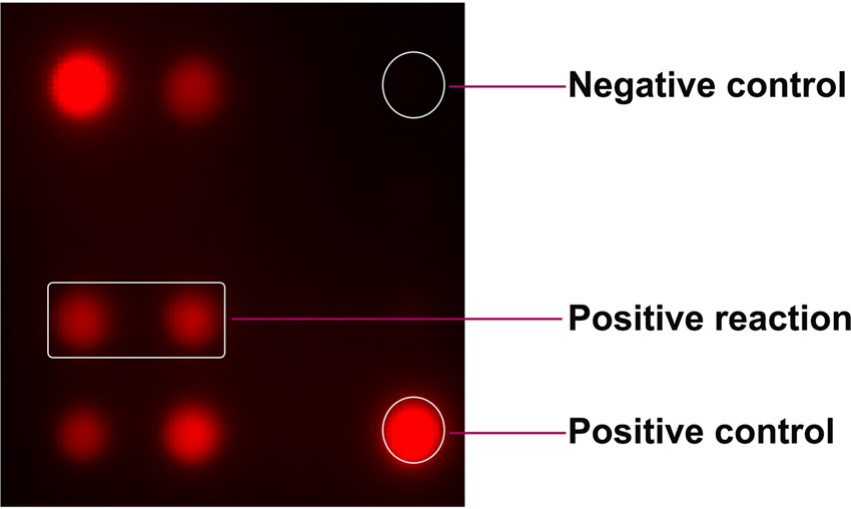
The peptide microarray for serum screening. All synthetic peptides were printed on iPDMS to form a 4-by-4 array. Chicken IgY was printed on two opposite corners (e.g., upper left corner and lower right corner) as the positive control, PBS was printed on the upper right corner as the negative control, and 13 peptides of H7 HA2 were printed on the remaining positions.

**FIG 2:**
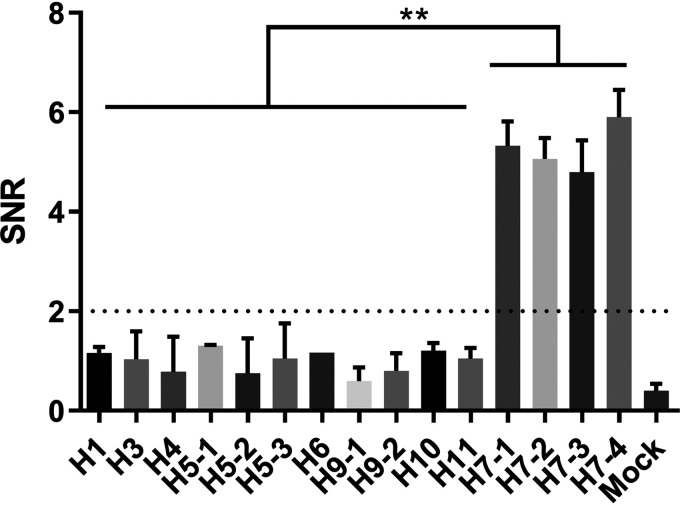
The signal-to-noise ratios (SNRs) of the reactions between antisera against different subtypes of AIVs and H7-12 peptides. Sera were diluted 1:100 (vol/vol) with PBS and screened with the peptide microarray to assay binding activity. SNRs were determined by using GenePix Pro 6.0 software. The dotted line represents an SNR of 2. Each reaction was repeated 3 times, and SNRs are expressed as mean values ± standard deviations. **, *P* < 0.01.

To screen H7N9 virus-specific epitopes, 13 peptides were synthesized and printed on iPDMS (modified silica gel film) to form a 4-by-4 peptide array with chicken IgY as the positive control and spotting buffer as the negative control (Fig. 1). Chicken antiserum samples against H1, H3, H4, H5, H6, H7, H9, H10, or H11 subtype AIVs (2 samples for each subtype) (Table 1) and 2 specific-pathogen-free (SPF) chicken serum samples were used for peptide screening. The results showed that the H7-12, H7-13, and H7-14 peptides showed positive binding activities to antisera against all H7 subtype AIVs (signal-to-noise ratio [SNR] of ≥2) (Fig. 2 and Table S1), among which the H7-13 peptide showed positive binding activities to antisera against H5, H6, and H11 subtype AIVs, the H7-14 peptide showed broad-spectrum binding activity to antisera against seven AIV subtypes but not to the H1 subtype, and only the H7-12 peptide showed negative binding activity to antisera against all other AIV subtypes (SNR of <2), indicating that the H7-12 peptide is a specific epitope for H7 subtype AIV.

**TABLE 1:** The AIV strains of different HA subtypes used in this study

Viruses	Subtype	HA titer (*n*log_2_)
A/Duck/Eastern China/103/03	H1N1	7
A/Duck/Eastern China/852/03	H3N2	6
A/Duck/Eastern China/160/02	H4N6	5
A/Mallard/Huadong/S/2005	H5N1	7
A/Chicken/Huadong/1111/16	H5N6	7
A/Chicken/Huadong/ZJ0104/16	H5N2	6
A/Duck/Eastern China/58/03	H6N2	7
A/Chicken/Shanghai/F/98	H9N2	9
A/Chicken/Fujian/SN/14	H9N2	6
A/Chicken/Huadong/RD5/13	H10N9	6
A/Duck/Eastern China/906/02	H11N2	7
A/Chicken/Jiangsu/JT/13	H7N9	8
A/Chicken/Jiangsu/JX05/14	H7N9	8
A/Chicken/Jiangsu/W1-8/15	H7N9	7
A/Chicken/Huadong/JD/17	H7N9	10
A/Chicken/Hebei/XT/2017	H7N9	9

**FIG 3:**
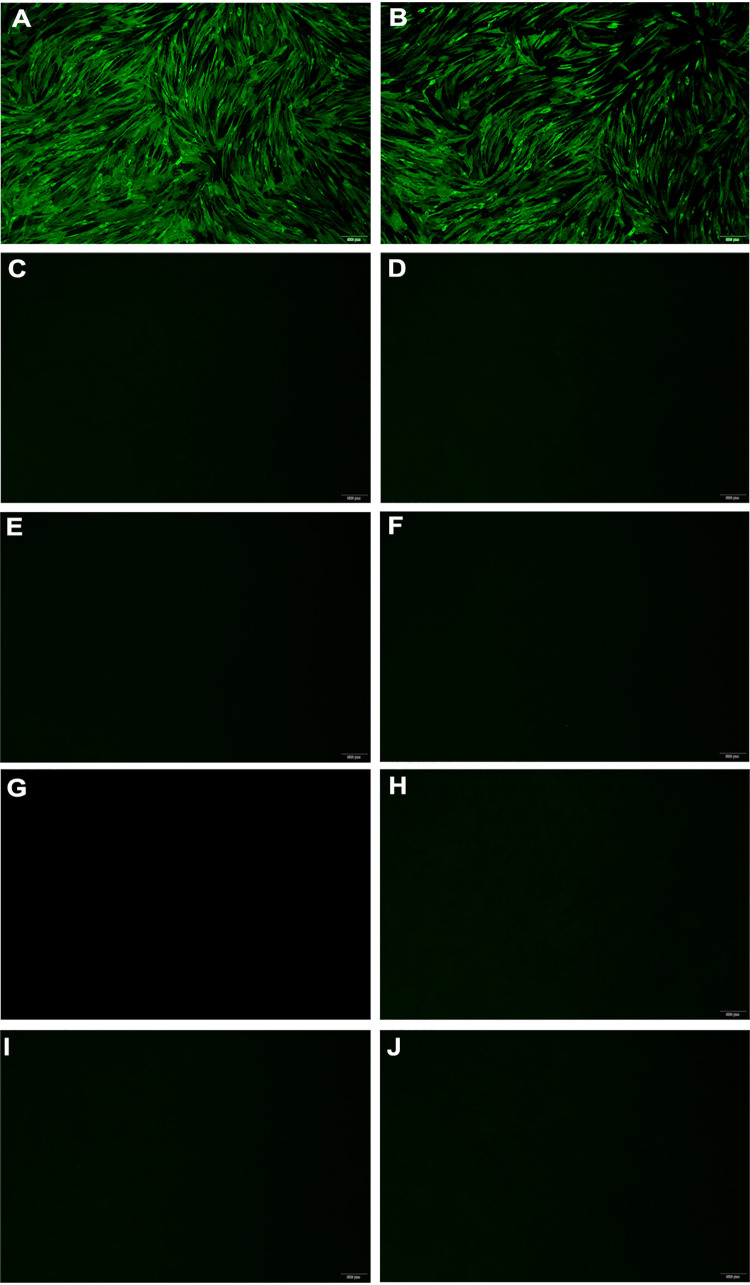
Identification of the epitope containing a short peptide specific for H7 subtype AIV by indirect immunofluorescence assay. CEF cells were infected with different subtypes of AIVs and fixed with methanol. After incubation with chicken antiserum against H7-12 peptide and FITC-conjugated goat anti-chicken IgY antibody, fluorescence was observed under a fluorescence microscope. (A to J) H7 AIV JD/17 with H7 AIV antiserum as positive control (A), H7 AIV JD/17 (B), H7 AIV JD/17 with unvaccinated SPF chicken serum as negative control (C), H1 AIV (D), H3 AIV (E), H4 AIV (F), H5 AIV (A/Chicken/Huadong/ZJ0104/16) (G), H6 AIV (H), H9 AIV (A/Chicken/Shanghai/F/98) (I), and H10 AIV (J). AIV strains are described in Table 1.

To confirm the specificity of the H7-12 peptide, H7-12 peptide conjugated with bovine serum albumin (BSA) was used to immunize chickens to prepare antipeptide serum. An immunofluorescence assay showed that green fluorescence was observed in H7 subtype AIV-infected chicken embryo fibroblast (CEF) cells treated with anti-H7-12 peptide antiserum, and no specific fluorescence was observed in CEF cells infected with other AIV subtypes, indicating that the H7-12 peptide is immunogenic and induces a specific antibody against H7 subtype AIV (Fig. 3).

### Generation of a recombinant H7N9 AIV with a chimeric HA gene.

**FIG 4:**
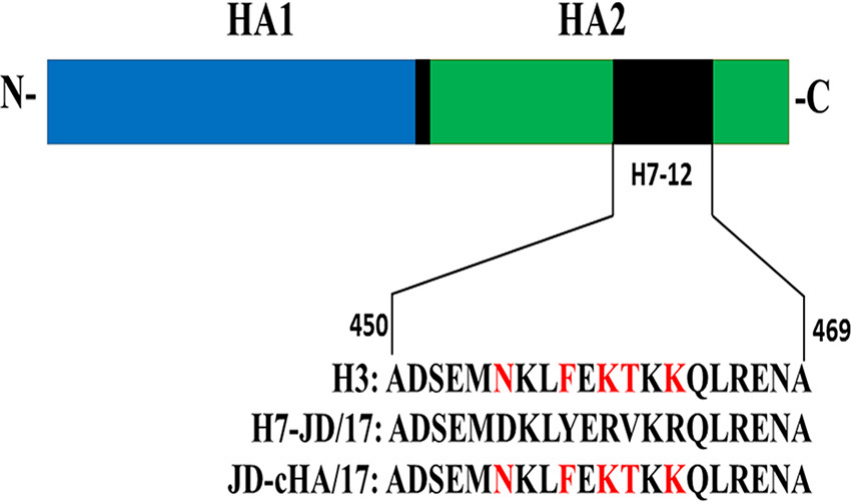
Structural schematic of JD-cHA/17 with chimeric HA. The amino acid sequence of the H7-12 peptide from JD/17 was replaced by that from H3 subtype AIV.

By sequence comparison, there was a 5- or 7-amino-acid difference between H3 or H1 AIV HA2 and the H7-12 peptide. The H7-12 peptides with different patterns in the GISAID database were analyzed, and it was found that an H7-12 peptide with the 5 amino acids of the H3 pattern does not exist in nature (0/2,878) (Table S2). To construct a DIVA vaccine candidate, the H7-12 peptide of HA2 in H7N9 AIV JD/17 was replaced with the peptide of H3N2 subtype AIV (Fig. 4). The rescued recombinant H7N9 AIV with the H3N2 H7-12 peptide was generated by reverse genetic technology and passaged for five generations in SPF chicken embryos. Sequence analysis showed that the recombinant H7N9 AIV had the predicted amino acids in the H7-12 peptide. There was no amino acid mutation in the HA protein after passage compared to the original HA protein sequence. The recombinant H7N9 AIV with chimeric HA was named JD-cHA/17 and subjected to the determination of biological features. The results showed that the JD-cHA/17 virus and the JD/17 parent strain had similar 50% egg infectious doses (EID_50_) (9.7 versus 9.3 log_10_ EID_50_/ml, respectively), 50% tissue culture infective doses (TCID_50_) (6.0 versus 6.0 log_10_TCID_50_/ml, respectively), and HA titers (10.0 versus 10.0 log_2_, respectively).

### Immunogenicity and protection of vaccine candidate strain JD-cHA/17.

**FIG 5:**
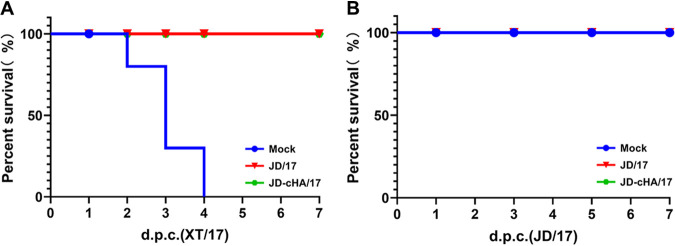
Survival rates of vaccinated chickens after HPAIV (A) or LPAIV (B) challenge. Red, green, and blue lines show the results for JD/17-, JD-cHA/17- and mock-vaccinated groups, respectively.

To test the immunogenicity of JD-cHA/17, SPF chickens were immunized once with JD-cHA/17 or its parental virus, JD/17. Three weeks after vaccination, chicken sera were collected to determine the mean hemagglutination inhibition (HI) antibody titers against homologous H7N9 LPAIV JD/17 and heterogenous H7N9 HPAIV XT/17. The HI antibody titers induced by JD-cHA/17 were similar to those induced by JD/17 (9.1 versus 8.9 log_2_, respectively, against JD/17 and 4.7 versus 5.1 log_2_, respectively, against XT/17) (Table 2), indicating the substitution of the H7-12 peptide of HA from H3N2 subtype AIV in JD/17 does not affect its immunogenicity. To test the immune protection of JD-cHA/17, vaccinated chickens were intranasally challenged with 10^6^ EID_50_ of either LPAIV JD/17 or HPAIV XT/17. Following the H7N9 LPAIV challenge, no obvious clinical symptoms were observed in infected chickens. The chickens in the group vaccinated with JD-cHA/17 showed no virus shedding in tracheal and cloacal swabs, while the chickens in the group vaccinated with JD/17 showed only 10% virus shedding in the trachea on day 3 postinfection. However, the chickens in the phosphate-buffered saline (PBS)-vaccinated group showed significantly higher virus shedding rates in tracheal and cloacal swabs, and even 100% virus shedding in the trachea on day 3 postinfection. For the HPAIV challenge, no obvious clinical signs were observed in the vaccinated chickens, but the chickens in the PBS group all died within 4 days postinfection (Fig. 5). At the same time, the chickens in the JD-cHA/17-vaccinated group showed no virus shedding, while the chickens in the JD/17-vaccinated group showed 10% virus shedding in the trachea on day 1 postinfection (Table 2), indicating that the JD-cHA/17 vaccine and the wild-type JD/17 vaccine induce similar rates of virus shedding protection against both LPAIV and HPAIV H7N9 challenge.

**TABLE 2:** Survival and virus shedding rates of vaccinated chickens postchallenge with H7N9 HPAIV or LPAIV

Vaccine	Mean HI titer ± SD (*n*log_2_) against:		Challenge virus	No. of chickens shedding virus/total no. in group at d.p.c.^*a*^:								% protection (no. of chickens non-shedding virus/total no. in group)
	Homologous virus	XT/17		1		3		5		7		
				Trachea	Cloaca	Trachea	Cloaca	Trachea	Cloaca	Trachea	Cloaca	
JD/17	8.8 ± 0.4	5.1 ± 0.8	JD/17	0/10	0/10	1/10	0/10	0/10	0/10	0/10	0/10	90 (9/10)
	9.1 ± 0.5	5.2 ± 0.7	XT/17	1/10	0/10	0/10	0/10	0/10	0/10	0/10	0/10	90 (9/10)
JD-cHA/17	9.0 ± 0.8	4.5 ± 1.3	JD/17	0/10	0/10	0/10	0/10	0/10	0/10	0/10	0/10	100 (10/10)
	9.2 ± 0.6	5.0 ± 0.6	XT/17	0/10	0/10	0/10	0/10	0/10	0/10	0/10	0/10	100 (10/10)
Mock (PBS)	ND^*b*^	ND	JD/17	8/10	1/10	10/10	6/10	9/10	6/10	3/10	0/10	
	ND	ND	XT/17	6/10	1/10	3/3	3/3	NS^*c*^	NS	NS	NS	
Control	ND	ND		0/5	0/5	0/5	0/5	0/5	0/5	0/5	0/5	

a d.p.c., day postchallenge.

b ND, no detection.

c NS, no survivors.

**FIG 6:**
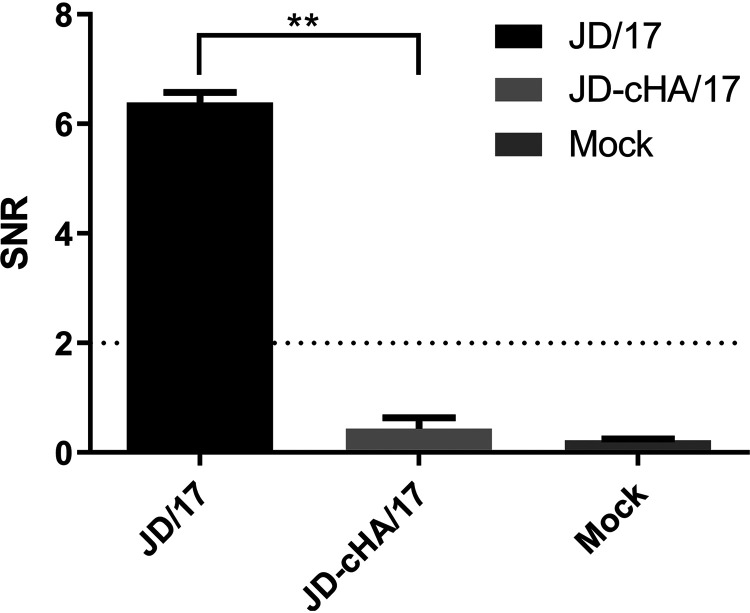
SNRs of immune sera from JD/17- or JD-cHA/17-vaccinated chickens (*n* = 5) detected by H7-12 peptide chip. Each reaction was repeated 3 times, and SNRs are expressed as mean values ± standard deviations. Unpaired Student’s two-sided *t* test was employed to determine the differences between the two groups. **, *P < *0.01.

To check whether the sera from infected chicken contained the anti-H7-12 peptide antibody, an H7-12 peptide microarray was used to test the JD/17-infected sera. The results showed that an HI antibody titer could be detected on day 7 postinfection and that the peak HI antibody titer was on day 28 postinfection. In contrast, the anti-H7-12 peptide antibody response was observed as early as day 3 postinfection (SNR of >2), and the peak anti-H7-12 peptide antibody response was on day 21 postinfection (Table 3), indicating that the H7-12 peptide microarray is sensitive in the detection of anti-H7-12 peptide antibody. When the H7-12 peptide microarray was used to test sera from immunized birds, none of the JD-cHA/17-vaccinated chickens showed a positive antibody response against H7-12 peptide on day 21 postvaccination, while high positive antibody responses were observed in sera from the parental JD/17-vaccinated chickens (Fig. 6) (*P < *0.01), suggesting that antibody induced by JD-cHA/17 can be discriminated from that induced by JD/17.

**TABLE 3:** HI titers and SNRs of sera from JD/17-infected chickens

Group, d.p.i.^*a*^	Mean value for infected sera ± SD (*n* = 5)	
	HI titer (nlog2)	SNR
Mock infected (PBS)	0	0.16 ± 0.07
JD/17 infected		
3	0	2.33 ± 0.15
5	0	2.49 ± 0.19
7	4.6 ± 0.5	3.53 ± 0.27
14	6.4 ± 0.5	4.63 ± 0.18
21	7.0 ± 0.6	4.69 ± 0.12
28	7.6 ± 1.4	4.69 ± 0.11

a d.p.i.: days postinfection.

## DISCUSSION

Several studies have proved that influenza viruses with chimeric HA can improve the characteristics of vaccine candidates. Hai et al. constructed functional influenza viruses with chimeric HA expressing a foreign globular head domain and part of the original HA1 and HA2 (28). Further study confirmed that the strategy could be applied to induce broad-based heterologous immunity and protection against various influenza virus subtypes by repeated immunization with chimeric HA constructs expressing the same stalk but an irrelevant head (29). Wang et al. generated a chimeric virus that contained the HA1 from the H1N1 A/California/7/2009 virus and the HA2 from the seasonal H1N1 A/South Dakota/6/3007 virus, which kept the immunogenicity and protection against H1N1 A/California/7/2009 virus and made HA cleavable by bromelain for antiserum production (30). In addition, recombinant H7H9 influenza vaccine with the H7 hemagglutinin transmembrane domain replaced by the H3 domain induces increased cross-reactive antibodies and improved interclade protection (31). However, these viruses were not designed for the DIVA strategy. Kim et al. constructed a chimeric H9/H5 recombinant vaccine that expressed the whole HA1 region of H9N2 subtype AIV and the HA2 region of H5N8 subtype HPAIV. Vaccination with the inactivated chimeric virus induced serum neutralizing antibodies against both H9 and H5 viruses but induced HI antibody only against H9 viruses, suggesting that the chimeric vaccine is suitable for a DIVA vaccine against H5 subtype AIV (32). However, the survival rates and virus shedding protection of the chimeric H9/H5 vaccine against H5 subtype AIV challenge were not comparable with those of wild-type H5 AIV inactivated vaccine. This is the first study to show that the generation of a chimeric HA H7N9 vaccine candidate by replacing an H7N9 AIV-specific epitope with that of H3 subtype AIV provides protection against H7N9 HPAIV and LPAIV challenge comparable to that of the wild-type-HA H7N9 AIV vaccine and induces no serum antibody against the H7N9 AIV-specific epitope detected by peptide microarray.

**FIG 7:**
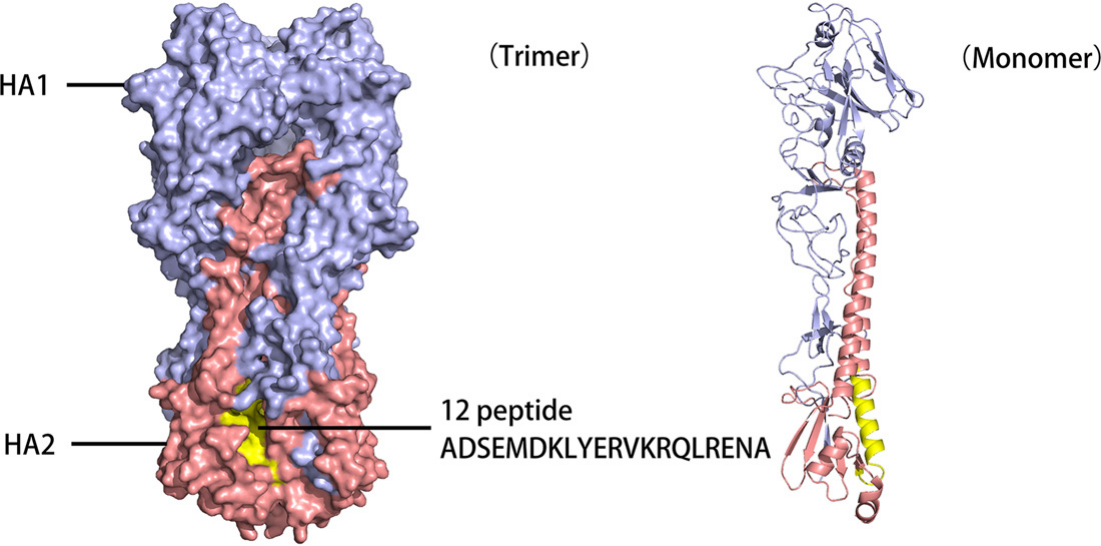
Locations of H7-12 peptide in simulated 3-D structure of H7N9 AIV HA protein. The position of the peptide on the HA molecule was analyzed using an X-ray crystal structure (Accession number: 6IDD) obtained from the Protein Database. The picture was generated by the SWISS-MODEL and the PyMOL system (https://pymol.org/2/).

To find specific epitopes for H7N9 AIV, a peptide microarray containing 13 peptides from H7N9 HA2 was subjected to serum screening. H7-14 peptide showed broad-spectrum binding activity to antisera against seven AIV subtypes but not to H1 subtype AIV, consistent with the results for a universal antigen epitope identified from H5 subtype AIV HA2 (33). Only the H7-12 peptide showed positive binding activity to the antisera against all H7 subtype AIVs but negative binding activity to the antisera against all other AIV subtypes. Further study confirmed that the antiserum against the H7-12 peptide only recognized H7N9 AIV and not other AIVs, suggesting that the H7-12 peptide is a specific epitope for H7 subtype AIV. In a simulated three-dimensional (3-D) structure of the HA open reading frame (Fig. 7), the H7-12 peptide consisted of α-helix and loop structures, which was exposed to the surface and able to induce antibody binding. The antibody against the H7-12 peptide in the sera collected from H7N9 AIV-infected chickens can be detected by peptide microarray as early as day 3 postinfection. In contrast, the HI antibody can be detected on day 7 postinfection, suggesting that the H7-12 peptide is immunogenic and antigenic. These results show that the peptide microarray may be a sensitive method for early detection of H7N9 AIV infection.

To construct DIVA vaccine candidates, H7N9 AIV JD/17 was selected as a reverse genetic backbone for its high HA or EID_50_ titers. The related H1 and H3 AIV peptides containing 7- or 5-amino-acid differences from the H7-12 peptide were selected to generate recombinant virus, but only the recombinant H7N9 virus containing the H3 peptide in place of the H7-12 peptide was successfully rescued, demonstrating that the HA-based structural design approach that replaces the H7-12 peptide with the H3 peptide containing the 5-amino-acid difference is readily applicable to generate recombinant influenza virus with chimeric HA. Our *in vitro* and *in vivo* studies revealed that the JD-cHA/17 recombinant virus possessed growth properties and immunogenicity similar to those of the wild-type H7N9 AIV JD/17, indicating that the replacement of the H7-12 peptide has no effect on the ability to produce HI antibodies against H7N9 AIVs and overcomes the biggest drawback of poor seroconversion for some DIVA vaccination strategies (27, 34–37).

Both the JD-cHA/17 and the JD/17 inactivated vaccine induced high HI titers against H7N9 AIV JD/17 on day 21 postvaccination, and they provided similar levels of virus shedding protection against LPAIV challenge (100% versus 90%). Both induced lower cross-reactive HI titers against H7N9 HPAIV XT/17, since there was only 94% amino acid homology between the LPAI and HPAI H7N9 AIVs. However, all vaccinated chickens acquired complete clinical protection against the H7N9 HPAIV challenge (data not shown), while nonvaccinated chickens died within 4 days. Moreover, no virus shedding was detected within 7 days postinfection in tracheal and cloacal swabs of JD-cHA/17-vaccinated chickens, comparable to the protection in JD/17-vaccinated chickens. These data indicated that the JD-cHA/17 vaccine with one dose in chickens provides protection against H7N9 HPAIV or LPAIV challenge, even in the case of antigenic drift between the vaccine and challenge strains. In addition, there was no antibody against the H7-12 peptide in the JD-cHA/17-vaccinated chickens, in contrast to positive antibody in the wild-type JD/17-vaccinated chickens, indicating the fitness of the JD-cHA/17 vaccine as an H7N9 AIV serological DIVA vaccine. Meanwhile, H7-12 peptide-based antibody can be detected as early as day 3 postinfection with the wild-type JD/17 virus, a better infection indicator than HI antibody for early diagnosis and disease control. Although there are 16 HA subtypes in AIVs, H3, H4, H7, H10, H14, and H15 AIVs are classified as one group based on phylogenetical analysis. We have confirmed no cross binding between the H7-12 peptide and the sera against H3, H4, or H10 AIV, and H14 or H15 AIVs are seldom isolated from chickens, indicating that the H7-12 peptide can be an H7N9 AIV-specific antigen for serological detection.

Several studies have demonstrated that poor seroconversion to the DIVA antigen can be detected when the birds are vaccinated and then infected (36–38) and that a high HI antibody response induced by avian influenza inactivated vaccine may contribute to clinical sign and virus shedding protection in vaccinated birds (39, 40). In our study, both the JD-cHA/17 and the JD/17 inactivated vaccine induced a high HI antibody response (about 9 log_2_), resulting in almost no virus shedding in tracheal and cloacal swabs of vaccinated chickens. Reduced virus replication in vaccinated chickens caused a weak DIVA response in serological detection and required sampling a higher number of birds to compensate for the lower seroconversion rate. Although antibody detection by an H7-12 peptide microarray was much more sensitive than that by HI test, the method needs to be further validated in infected chickens with different HI antibody levels. Also, an H7-12-based enzyme-linked immunosorbent assay (ELISA) may be developed for further use in the field. In addition, H7N9 AIV undergoes rapid evolution and antigenic drift in China (5), so by now, the H7N9 avian influenza vaccine strain has been updated to Re-3 of H7N9 AIV. Accordingly, the HA gene of a new vaccine strain can be modified as a chimeric HA by substitution of the H3 AIV short peptide to meet the needs of vaccine updating.

In summary, we identified an epitope containing a short peptide (ADSEMDKLYERVKRQLRENA) that is highly specific for H7N9 AIV and constructed an AIV strain named JD-cHA/17 with a chimeric HA by substitution of the H3 subtype AIV epitope. The JD-cHA/17 strain had replication characteristics and immunogenicity similar to those of the wild-type strain, JD/17. The chickens vaccinated with JD-cHA/17 vaccine produced similar levels of HI antibody but no antibody against the H7-12 peptide, as detected by an H7-12 peptide microarray, and the JD-cHA/17 vaccine provided similar levels of clinical protection and significantly reduced virus shedding against LPAI or HPAI H7N9 virus challenge compared with the chickens vaccinated with wild-type JD/17 vaccine. This DIVA vaccine design provides a novel strategy for H5 or H7 subtype avian influenza eradication.

## MATERIALS AND METHODS

### Ethical approval.

Virus preparation and titration, all animal studies, and serological testing for chimeric H7/H3 and H7N9 viruses were performed in an animal biosafety level 3 (ABSL-3) facility at Yangzhou University and were approved by The Jiangsu Administrative Committee for Laboratory Animals (permit number SYXK-SU-2016-0020) according to the guidelines for laboratory animal welfare and ethics of the Jiangsu Administrative Committee for Laboratory Animals.

### Virus and cells.

A total of 14 strains of AIVs (Table 1) isolated from live poultry markets, including nine different AIV subtypes (H1, H3, H4, H5, H6, H7, H9, H10, and H11), were used to prepare sera. An H7N9 HPAIV, A/Chicken/Hebei/XT/2017 (H7N9) (XT/17), was isolated from diseased chickens. All viruses were propagated in SPF embryonated chicken eggs. 293T and Madin-Darby canine kidney (MDCK) cells were grown in Dulbecco’s modified Eagle’s medium (DMEM; HyClone, USA) supplemented with 10% fetal bovine serum (FBS; Gibical, USA) plus antibiotics. Primary chicken embryo fibroblast (CEF) cells were prepared from 9- to 10-day-old SPF chicken embryos and cultured in M199 medium (HyClone, USA) with 4% FBS. The cells were incubated at 37°C with 5% CO_2_.

### Peptide microarray.

According to the deduced amino acid sequence of the HA2 protein of the H7N9 LPAI virus JD, a total of 13 overlapping peptides (10 amino acids overlapped between two adjacent peptides) were synthesized by GL Biochem Ltd. (Shanghai, China), except for the failure of the 5th and 11th peptides (Table 4). Synthetic peptides were sampled on iPDMS (modified silica gel film; SJ Biomaterials, Suzhou, China), and the workflow of the microarray was mainly based on that of the previous study (41). Sera were diluted 1:100 (vol/vol) with PBS, and a 200-μl dilution was added to each well of the chip. The chip was incubated on a shaker for 2 h (150 rpm and 4°C) and subsequently washed three times with TBST (20 mM Tris base, pH 6.8, 137 mM NaCl, 0.1% Tween 20). Following incubation with 200 μl of 1:25,000-diluted horseradish peroxidase (HRP)-labeled goat anti-chicken IgY (Abcam, Cambridge, United Kingdom) in dilution buffer (Guardian peroxidase conjugate stabilizer/diluent; Thermo Fisher Scientific, USA) for an additional 1 h at 37°C and three washes with TBST, 15 μl chemiluminescent substrate (SuperSignal West Pico Plus chemiluminescent substrate; Thermo Fisher, USA) was added to each well of the chip. Chemiluminescent signals were captured by a charge-coupled device (CCD) camera (LAS4000 imaging system; GE Healthcare Life Sciences, USA) and saved as images in TIFF format. After that, the chemiluminescence intensity of each peptide point and the background were converted into signal-to-noise ratios (SNRs) using GenePix Pro 6.0 software. An SNR of ≥2 was identified as seropositive.

**TABLE 4:** Sequences of the 13 peptides of H7N9 HA2 investigated in this study

Peptide	Position	Sequence
H7-1	340–359	GLFGAIAGFIENGWEGLIDG
H7-2	350–369	ENGWEGLIDGWYGFRHQNAQ
H7-3	360–379	WYGFRHQNAQGEGTAADYKS
H7-4	370–389	GEGTAADYKSTQSAIDQITG
H7-6	390–409	KLNRLIAKTNQQFELIDNEF
H7-7	400–419	QQFELIDNEFNEVEKQIGNV
H7-8	410–429	NEVEKQIGNVINWTRDSITE
H7-9	420–439	INWTRDSITEVWSYNAELLV
H7-10	430–449	VWSYNAELLVAMENQHTIDL
H7-12	450–469	ADSEMDKLYERVKRQLRENA
H7-13	460–479	RVKRQLRENAEEDGTGCFEI
H7-14	470–489	EEDGTGCFEIFHKCDDDCMA
H7-15	480–499	FHKCDDDCMASIRNNTYDHR

### Serum preparation.

Twenty-one-day-old SPF chickens (Meiliya-Weitong Experimental Animal Technology Co., Ltd., Beijing, China) were housed in cages with *ad libitum* access to food and water. Five chickens in each group were immunized with inactivated AIVs with oil emulsion adjuvants or BSA-conjugated peptides (GL Biochem Ltd., China) with Freund’s adjuvant (Sigma, USA) and boosted at a 3-week interval. Chickens were euthanized by manual cervical dislocation at 2 to 3 weeks after the second vaccination, and their sera were collected and identified by hemagglutination inhibition (HI) assay for antisera generated by whole virus and by peptide microarray for antisera generated by peptide conjugate.

### Hemagglutination inhibition assay.

The serum samples were serially diluted 2-fold in PBS in a 25-μl volume in a 96-well plate, and 4 hemagglutination units in 25 μl of A/Chicken/Huadong/JD/2017 (JD) virus was added to each well. After 30 min of incubation at room temperature, 50 μl of 1% chicken erythrocytes diluted in PBS was added to each well and the plate incubated for another 30 min. HI titers were calculated as the reciprocal of the highest serum dilution that inhibited hemagglutination by binding with the virus.

### Immunofluorescence assay.

A monolayer CEF cell culture in a 96-well plate was infected by different AIV subtypes at a multiplicity of infection (MOI) of 10, with 1 μg/ml TPCK (l-1-tosylamido-2-phenylethyl chloromethyl ketone)-trypsin (Sigma-Aldrich, USA) as needed. After 12 h of incubation at 37°C with 5% CO_2_, the cells were washed and fixed in precooled methanol (−20°C). Next, the cells were incubated with antipeptide chicken antiserum at a dilution of 1:1,000 for 1.5 h at 37°C and further incubated with fluorescein isothiocyanate (FITC)-conjugated goat anti-chicken IgY antibody (Abcam, Cambridge, United Kingdom) at a dilution of 1:500 for another 1 h. Finally, the fluorescence staining was visualized with an IX71 fluorescence microscope (Olympus, Japan).

### Construction of recombinant H7N9 AIV with a chimeric HA gene.

Eight gene segments (PB2, PB1, PA, HA, NP, NA, M, and NS) of JD/17 were amplified by reverse transcription-PCR (RT-PCR) and cloned into the pHW2000 vector. The HA gene of JD/17 virus with the substitution of the H3 subtype virus HA2-12 peptide was generated by overlapping PCR using two primer pairs (forward, GACCTCCGAAGTTGGGGGGGAGCAAAAGCAGGGGATACAAAATGA; internal forward, TGAACGAAATTTTTAAAATGCAGCTGAGAGAGAATGCTGA; internal reverse, AAAAATTTCGTTCAGTTTCAACATTTCTGAATCAGCCAGA; and reverse, GCATTTTGGGCCGCCGGGTTATTAGTAGAAACAAGGGTGTTTTTTCCA) and also cloned into the pHW2000 vector. The resulting plasmids were confirmed by sequencing to verify the construction. The recombinant virus was rescued via an 8-plasmid reverse genetics technology as described previously (42). A mixture of 293T and MDCK cells was cotransfected with the above-mentioned eight plasmids using PolyJet transfection reagent (SignaGen, USA). At 60 to 72 h posttransfection, the cells and supernatant were harvested and inoculated into 10-day-old SPF embryonated chicken eggs to propagate the rescued virus. The rescued virus was confirmed by sequence analysis and named JD-cHA/17. The EID_50_ of the JD-cHA/17 virus was determined and calculated according to the Reed-Muench method (43).

### Vaccination and challenge in chickens.

Inactivated vaccine was prepered as follows. Virus in the allantoic fluids of eggs infected with JD or JD-cHA/17 (10^8.5^ EID_50_/ml) was inactivated by formalin (0.1% final concentration) (44, 45), and then mixed with a water-in-oil white oil adjuvant (Sinopharm Yangzhou Weike Biological Engineering Co. Ltd.) in a ratio of 1:3 (vol/vol) to for emulsifying a water-in-oil form prepare a vaccine. Twenty-one-day-old SPF White Leghorn chickens purchased from Beijing Meiliya-Weitong Experimental Animal Technology Co., Ltd. were randomly divided into six challenge groups (10 chickens per group) and one control group (5 chickens). Chickens in challenge groups were vaccinated by subcutaneous injection of 0.3 ml of JD or JD-cHA/17 vaccine preparation or PBS. The serum samples were collected from all chickens at 3 weeks postvaccination. After serum sample collection, the chickens were inoculated intranasally with 10^6^ EID_50_ of JD/17 or XT/17 in a 0.1-ml volume. Tracheal and cloacal swabs were collected from chickens at 1, 3, 5, and 7 days postchallenge (d.p.c.) and resuspended in 1 ml PBS for virus titrations. Virus shedding was determined by inoculation of 10-day-old SPF embryonated chicken eggs with treated swab fluids and hemagglutination assay with chicken embryo allantoic liquid.

In addition, five chickens were inoculated intranasally with 10^6^ EID_50_ of JD/17 in a 0.1-ml volume to mimic the natural infection of the LPAI H7N9 virus. Serum samples were collected at 0, 3, 5, 7, 14, 21, and 28 d.p.c. for HI assay and peptide microarray.

### Statistical analysis.

The SNRs of the immune serum reactions were analyzed by unpaired Student’s two-sided *t* test. *P* values of <0.05 were regarded as showing statistically significant differences.
