# Supplementary material for: Development of an Inactivated H7N9 Subtype Avian Influenza Serological DIVA Vaccine Using the Chimeric HA Epitope Approach
Source: Microbiol Spectr. 2021 Sep 29;9(2):e00687-21. doi: 10.1128/Spectrum.00687-21 (PMC8557892; doi:10.1128/Spectrum.00687-21)
Supplement: SUPPLEMENTAL FILE 1 — Supplemental material. Download SPECTRUM00687-21_Supp_1_seq2.pdf, PDF file, 0.2 MB [file spectrum00687-21_supp_1_seq2.pdf]

Table S1. The SNRs of sera from vaccinated chicken with different subtypes of AIV detected by peptide microarray

Serotype	SNRs (Mean±SD)												
	**H7-1**	**H7-2**	**H7-3**	**H7-4**	**H7-6**	**H7-7**	**H7-8**	**H7-9**	**H7-10**	**H7-12**	**H7-13**	**H7-14**	**H7-15**
	**340-359aa**	**350-369aa**	**360-379aa**	**370-389aa**	**390-409aa**	**400-419aa**	**410-419aa**	**420-439aa**	**430-449aa**	**450-469aa**	**460-479aa**	**470-489aa**	**480-499aa**
Mock	0.08±0.04*	0.44±0.27	0.61±0.1	0.15±0.04	0.11±0.02	0.08±0.05	0.05±0.01	0.61±0.01	0.23±0.08	0.21±0.11	0.07±0.03	0.16±0.06	0.35±0.08
A/Duck/Eastern China/103/03 (H1N1)	4.39±1.5	1.15±0.27	0.4±0.03	1.48±0.24	0.08±0.02	0.90±0.02	2.24±0.41	1.31±0.1	0.96±0.39	1.16±0.09	1.29±0.38	1.63±0.5	0.26±0.08
A/Duck/Eastern China/852/03 (H3N2)	1.51±0.23	1.94±0.06	0.85±0.6	0.67±0.35	1.46±0.15	0.50±0.44	2.58±1.27	0.87±0.39	2.40±0.3	1.03±0.4	0.66±0.19	3.09±0.15	0.41±0.14
A/Duck/Eastern China/160/02 (H4N6)	1.35±0.34	2.38±0.51	0.23±0.02	1.03±0.7	1.27±0.92	4.42±3.96	4.79±2.39	7.00±1.66	1.08±0.68	0.78±0.5	0.43±0.05	2.34±0.81	6.50±0.98
A/Mallard/Huadong/S/2005 (H5N1)	0.81±0.04	2.27±0.95	0.83±0.05	1.02±0.54	1.04±0.28	2.30±0.16	2.32±0.79	3.42±0.16	8.51±1.37	1.31±0.01	0.02±0.02	3.92±2.38	1.49±0.22
A/Chicken/Huadong/1111/16 (H5N6)	0.74±0.22	3.89±0.29	0.93±0.06	2.37±0.3	2.49±0.24	0.93±0.61	3.19±0.04	0.73±0.58	0.34±0.11	0.75±0.5	2.75±0.69	2.13±0.25	0.95±0.2
A/Chicken/Huadong/ZJ0104/16 (H5N2)	0.45±0.18	0.91±0.65	0.48±0.35	0.73±0.3	1.52±1.15	1.17±0.29	3.47±2.44	0.20±0.02	1.41±0.09	1.05±0.5	0.16±0.03	2.41±1.34	2.22±1.64
A/Duck/Eastern China/58/03 (H6H2)	1.4±0.39	3.29±0.06	0.54±0.01	0.89±0.01	1.29±0.16	0.54±0.23	1.30±0.33	4.24±0.56	0.12±0.03	1.17±0	3.77±1.39	3.43±0.03	1.74±0.64
A/Chicken/Jiangsu/JT/13 (H7N9)	7.21±0.58	6.81±0.68	0.10±0.04	2.40±1.92	0.98±0.07	3.67±2.21	0.73±0.2	0.82±0.25	0.79±0.27	5.32±0.35	5.72±0.54	2.47±0.37	1.01±0.06
A/Chicken/Jiangsu/JX05/14 (H7N9)	5.81±0.19	1.65±0.21	1.52±0.38	0.83±0.04	0.98±0.68	1.5±1.14	1.74±0.55	0.73±0.33	1.58±0.47	5.06±0.3	4.30±1.06	3.20±0.75	0.51±0.02
A/Chicken/Jiangsu/W1-8/15 (H7N9)	1.64±0.35	2.33±0.12	7.68±1.1	0.92±0.3	1.13±0.43	3.05±1.87	2.11±0.37	1.79±0.58	2.41±0.24	4.8±0.45	2.92±1.19	4.17±0.08	1.31±0.56
A/Chicken/Huadong/JD/17 (H7N9)	3.12±0.86	3.93±0.67	0.93±0.06	0.95±0.47	2.26±0.4	2.55±0.15	4.87±3.44	2.47±0	1.36±0.3	5.90±0.39	4.85±2.12	3.11±0.74	1.09±0.01
A/Chicken/Shanghai/F/98 (H9N2)	3.78±0.86	2.21±0.62	3.89±0.64	0.67±0	0.86±0.33	1.29±0.83	2.98±0.04	0.78±0.12	2.12±0.1	0.59±0.2	1.30±0.28	8.53±1	1.10±0.24
A/Chicken/Fujian/SN/14(H9N2)	2.11±0.45	1.85±0.16	1.57±0.45	2.12±0.14	1.57±0.09	1.92±0.65	1.77±0.1	1.15±0.09	1.76±0.16	0.8±0.25	1.15±0.1	6.14±0.2	1.90±0.26
A/Chicken/Huadong/RD5/13 (H10N9)	3.31±0.96	1.94±0.72	0.42±0.02	0.85±0.31	1.13±0.27	0.86±0.13	1.50±0.14	1.67±0.23	0.29±0.19	1.21±0.11	0.90±0.2	9.54±4.67	0.95±0.32
A/Duck/Eastern China/906/02 (H11N2)	2.02±0.35	1.08±0.01	0.67±0.37	1.44±0.36	1.15±0.11	0.76±0.16	2.56±0.97	1.35±0.32	0.25±0.12	1.05±0.15	5.35±0.52	3.86±2.28	0.75±0.05

*cut-off values. SNRs ≥2 is positive, SNRs < 2 is negative.

Table S2 The proportion of the 12 peptide with different patterns in H7N9 AIV

H7N9	H7-12 peptide	H3 insert						others	Sample size
		468N	471F	473K	474T	476K	468N-476K		
LPAIV	2484	115	0	0	0	47	0	22	2668
HPAIV	167	30	0	0	0	10	0	3	210
sum	2651	145	0	0	0	57	0	25	2878
